# Abnormal Uterine Bleeding With Leiomyomas: A Case Report of Its Natural Course and Therapeutic Management

**DOI:** 10.7759/cureus.69153

**Published:** 2024-09-11

**Authors:** Samreen Ahmed, Saba Ahmed, Mansoor Gullabzada, Uzma Gullabzada, Ketan Jobanputra

**Affiliations:** 1 Medicine, Saint James School of Medicine, Arnos Vale, VCT; 2 Cardiology, Washington University of Health and Sciences, San Pedro, BLZ; 3 Obstetrics and Gynecology, UChicago Medicine AdventHealth, Bolingbrook, USA

**Keywords:** abnormal uterine bleeding, heavy menstrual bleeding, hysterectomy, leiomyoma, myfembree, pelvic pain, personalized treatment, pharmacological treatment, supracervical hysterectomy, uterine fibroids

## Abstract

Uterine fibroids (UFs), or leiomyomas, are common benign tumors affecting a significant proportion of women by the age of 50 years. While typically non-cancerous, UFs can severely impact the quality of life (QoL) through symptoms such as heavy menstrual bleeding (HMB) and pelvic pain. We report a case of a 45-year-old Hispanic female with a history of hypertension, diabetes, nephrolithiasis, and a solitary kidney, who presented with symptomatic UFs and an ovarian cyst. Despite initial pharmacological treatment with Ortho Micronor and Myfembree, the patient experienced persistent symptoms, prompting surgical intervention. A supracervical hysterectomy, bilateral salpingectomy, lysis of adhesions, and left ovarian cystectomy were performed, resulting in symptom relief and benign postoperative findings. This case report highlights the need for personalized treatment plans and comprehensive patient counseling to manage complex gynecological conditions effectively.

## Introduction

Uterine fibroids (UFs), also known as leiomyomas, are the most prevalent benign tumors in women worldwide, with a prevalence of ≥80% in all women by the age of 50 years [1]. Fibroids are nodular neoplasms of uterine smooth muscle cells with abundant extracellular matrix (ECM) [2]. In rare cases, leiomyomas are considered malignant or progress to malignancy [3]. UFs vary in size, composition, and number among patients. For this reason, symptoms manifest differently across individuals but still impact their quality of life (QoL) and daily activities.

Heavy menstrual bleeding (HMB) or abnormal uterine bleeding (AUB), is the most common symptom of leiomyomas. It occurs in about one-third of UF patients and can lead to life-threatening anemia [1]. Pelvic pain is another frequent problem reported by patients [4]. Moreover, submucosal leiomyomas notably cause menorrhagia, infertility, miscarriage, and preterm birth, [5] as well as “bulk” symptoms such as increased pelvic pressure, urinary frequency, and constipation as they compress nearby organs [6]. Leiomyomas are further associated with preterm delivery, spontaneous abortion, and cesarean delivery [7].

The diagnosis of leiomyomas largely depends on clinical judgment but ultrasonography is the most recommended imaging technique for fibroids in the United States. Management options for leiomyomas are customized to the individual patient based on the various symptoms, patient’s age, fibroid size, location, and patient’s wish for fertility preservation [8]. This report aims to explore the challenges and decision-making processes involved in treating symptomatic UFs.

## Case presentation

A 45-year-old Hispanic gravida 2 and para 2 female with a past medical history of hypertension, diabetes mellitus II, nephrolithiasis, and solitary kidney was referred to the clinic by her primary care physician for an ovarian cyst and AUB. In December 2022, the patient had an initial consultation when she reported experiencing bilateral pelvic pain, irregular menstrual cycles, and heavy bleeding that had necessitated changing five to six fully soaked pads daily. Despite being prescribed norethisterone (Ortho Micronor 0.35 mg) by her primary care provider, she continued to have AUB. Pelvic examination showed an enlarged non-tender firm uterus.

Pelvic ultrasound showed a 10-12-week enlarged and irregular uterus with dimensions of 76 x 60 x 48 mm. The endometrium thickness was measured at 9.16 mm, the right ovary at 24 x 23 x 21 mm, the left ovary at 17 x 21 x 11 mm, and left ovary simple cyst 34 at mm; fibroid #1 measured 11.6 x 14 mm in the anterior mid-position, fibroid #2 measured 17.4 x 15.2 mm in the intramural mid-position, fibroid #3 measured 22.2 x 20.6 mm in the anterior position, and fibroid #4 measured 34.4 x 17 mm in the anterior position. Due to a suboptimal pelvic ultrasound, further diagnostic studies were conducted, including a hysterosonogram and an endometrial biopsy. Results showed no endometrial polyp and benign proliferative endometrium with focal stromal and/or glandular breakdown. Tumor marker alpha-fetoprotein (AFP) and cancer antigen (CEA)-125 were within normal limits: 3 ng/mL and 16 Units/mL, respectively.

The patient was counseled on her conditions of leiomyoma, AUB, and ovarian cyst. Given that she had been using norethisterone (Ortho Micronor 0.35 mg) but still experienced AUB, she was prescribed Myfembree. The patient returned for a follow-up appointment after six months on Myfembree. She reported that her menstrual cycles had become more regular over the past six months, with each cycle lasting 30 days and bleeding occurring for 6-10 days. She changed her pads seven times a day at the start of her period, decreasing in frequency after day three. However, she continued to experience bilateral adnexal pain, which she described as constant and stabbing, rating it as 6-10 out of 10. She noted that Tylenol only provided mild relief. 

In March 2024, the patient returned with complaints of increased pelvic pain and pressure. She reported that the pain had worsened, significantly impacting her daily activities. She described the pain as constant, rating it as 7 out of 10, with occasional sharp exacerbations, which she rated as 10 out of 10. Although she had initially experienced some improvement with Myfembree, her menstrual periods had since lengthened to about seven days, with a heavy flow. Despite these symptoms, her hemoglobin and mean corpuscular volume remained within the normal range at 13.9 g/dL and 86.8 fL, respectively. Due to the ineffectiveness of pharmacotherapy and worsening symptoms, the patient requested a hysterectomy. The surgical options discussed included a supracervical hysterectomy, a possible total abdominal hysterectomy, and a potential bilateral salpingo-oophorectomy.

Preoperative pelvic ultrasound revealed changes in the size of the fibroids: fibroid #1 measured 20.9 x 19.9 mm in the anterior mid-position, fibroid #2 measured 10.5 x 14.1 mm in the intramural mid-position, fibroid #3 measured 14.5 x 12.6 mm in the anterior position, and fibroid #4 measured 13.2 x 12.8 mm in the anterior position. The endometrium thickness was measured at 9.22 mm. Ovarian tumor markers were within normal limits (Table 1).

**Table 1:** Tumor and pregnancy markers AFP: alpha-fetoprotein; CA 19-9: cancer antigen 19-9; CA 125: cancer antigen 125; CEA: carcinoembryonic antigen; hCG: human chorionic gonadotropin; LDH: lactate dehydrogenase

Test	Patient value	Reference range
CEA	1.4	0.0-4.7 ng/mL
AFP	2.2	0.0-6.4ng/mL
CA 19-9	12	0.0-6.4 ng/mL
CA 125	22.2	0.0-38.1 U/mL
hCG, beta subunit, Qnt	<1	0-5 mIU/mL (non-pregnant)
LDH	154	119-226 IU/L

**Figure 1:**
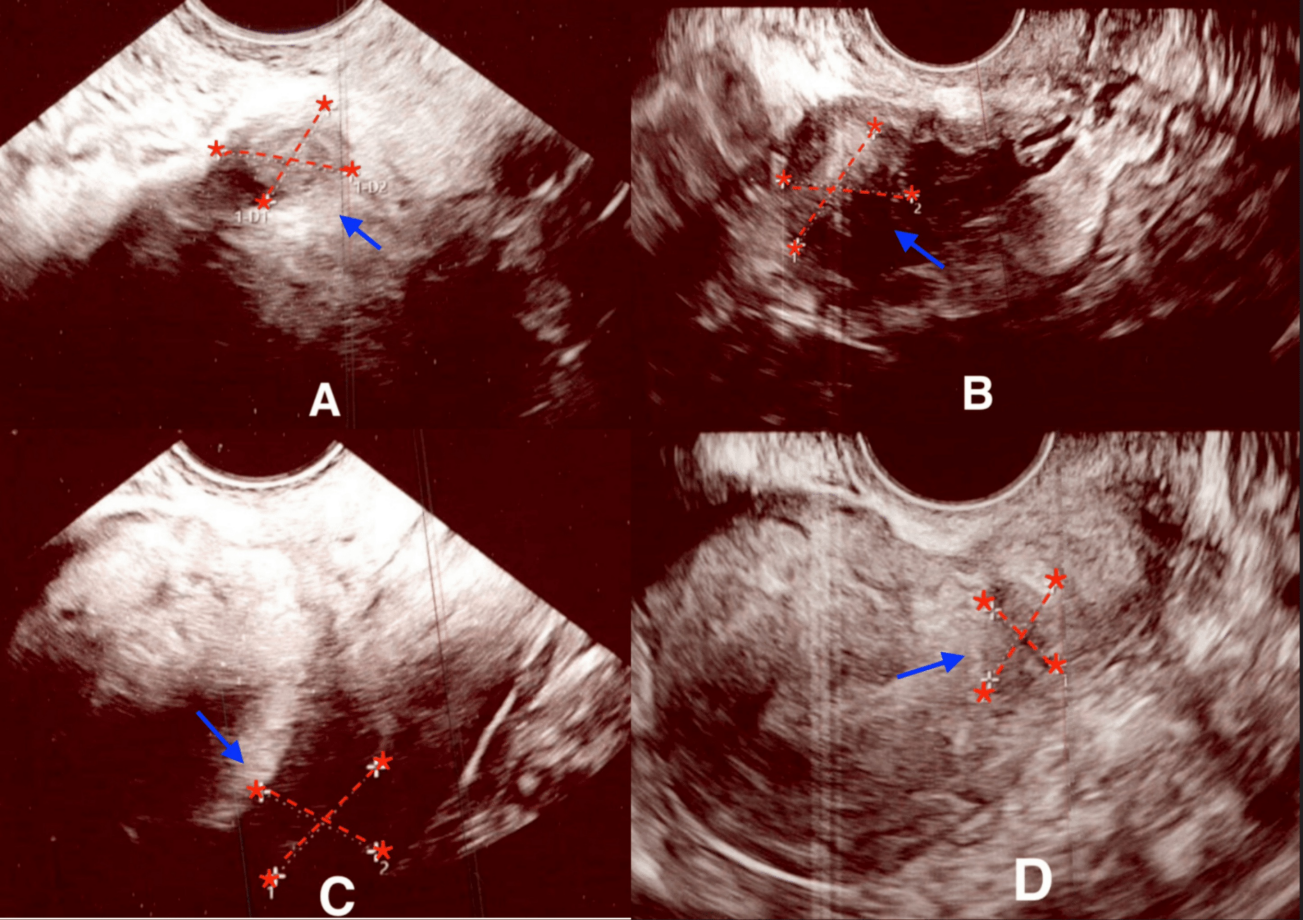
Transvaginal ultrasound images showing comparison of fibroid's size following norethisterone vs. Myfembree therapy (A) Fibroid #1 measured 11.6 x 14 mm after treatment with norethisterone. (B) Fibroid #1 measured 20.9 x 19.99 mm after treatment with Myfembree. (C) Fibroid #2 measured 17.4 x 15.2 mm after treatment with norethisterone. (D) Fibroid #2 measured 10.5 x 14.1 mm after treatment with Myfembree

**Figure 2:**
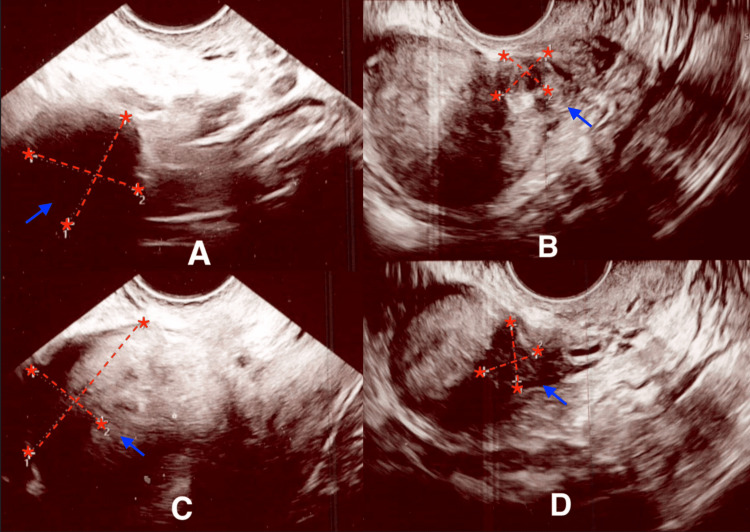
Transvaginal ultrasound images showing comparison of fibroid's size following norethisterone vs. Myfembree therapy (A) Fibroid #3 measured 22.2 x 20.6 mm after treatment with norethisterone. (B) Fibroid #3 measured 14.5 x 12.6 mm after treatment with Myfembree. (C) Fibroid #4 measured 34.4 x 17 mm after treatment with norethisterone. (D) Fibroid #4 measured 13.2 x 12.8 mm after treatment with Myfembree

**Figure 3:**
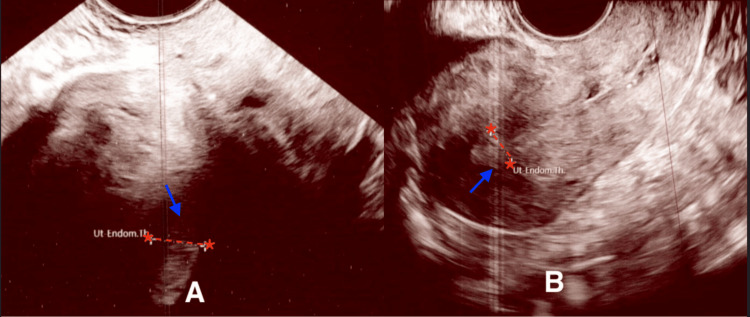
Transvaginal ultrasound images showing comparison of endometrial thickenss following norethisterone vs. Myfembree therapy (A) Endometrial thickness measured 9.16 mm after treatment with norethisterone. (B) Endometrial thickness measured 9.22 mm after treatment with Myfembree

Moreover, CT abdominal pelvis without IV contrast showed an increase in the size of low attenuation adnexal mass lesion of 3 x 8.2 cm in AP and transverse dimensions compared to the previous measurement of 3.1 x 2.4 cm. Ultrasound images in Figures 1-3 compare the fibroid sizes and endometrial thickness post-norethisterone (Ortho Micronor 0.35 mg) and Myfembree therapy.

In May 2024, the patient underwent a supracervical hysterectomy, bilateral salpingectomy, lysis of adhesions, and left ovarian cystectomy. The procedure was completed without any complications. Histological analysis of the specimens showed a benign serous cystadenoma on the left ovarian wall, fallopian tubes with no significant abnormalities, secretory phase endometrium, and leiomyoma, with no signs of malignancy in the uterus. During follow-up, the patient’s incision site was found to be healing well. She reported no issues except for mild pelvic pain, which she rated as 4 out of 10.

## Discussion

We describe the clinical course of a 45-year-old Hispanic female with complex gynecological symptoms, including HMB, pelvic pain, UFs, and an ovarian cyst, compounded by a history of hypertension, type II diabetes, nephrolithiasis, and a solitary kidney. AUB is a prevalent condition affecting 10-30% of women of reproductive age. The International Federation of Gynecology and Obstetrics defined the most common causes of abnormal uterine bleeding using the acronym PALM-COEIN [9]. The PALM group (polyp, adenomyosis, leiomyoma, malignancy, and hyperplasia) consists of structural causes that can be identified via imaging or biopsy. The COEIN group (coagulopathy, ovulatory dysfunction, endometrial, iatrogenic, and not otherwise classified) includes nonstructural causes. These categories are not mutually exclusive, and patients may experience multiple causes simultaneously [10]. 

Our patient reported irregular menstrual cycles and heavy bleeding, requiring frequent pad changes. Initial imaging with pelvic ultrasound showed an enlarged uterus with multiple fibroids and a thickened endometrium, while further studies, including a hysterosonogram and endometrial biopsy, confirmed benign proliferative endometrium without polyps. Tumor markers (AFP and CA 125) were normal. All these findings were consistent with a diagnosis of UFs.

UFs, also known as leiomyomas, are the most prevalent non-cancerous tumors in women worldwide, impacting up to 70% of women of reproductive age and accounting for about 30% of all hysterectomies. They are known to cause symptoms such as HMB and pelvic pain [11]. Various theories have been proposed in the literature to explain the relationship between UFs, AUB, and HMB. The pathophysiology behind AUB in the presence of structural conditions like UFs or adenomyosis remains unclear. There is an ongoing debate about whether excessive bleeding is directly caused by UFs or is the result of preexisting abnormalities in the endometrium, such as a "secondary endometrial disorder".

UFs can lead to changes in the vascular structure and function of the endometrium, which in turn stimulates the production of angiogenic factors that promote increased blood vessel formation [12]. Factors like vascular endothelial growth factor, platelet-derived growth factor, and endothelin-1 play a role in this process. Other contributors to AUB and HMB in the presence of UFs include the expanded endometrial surface area, enlarged uterine cavity, or dilated blood vessels on fibroid surfaces [13]. The abnormal angiogenesis, which may involve disrupted vessel maturation, leads to the formation of immature and fragile blood vessels [14]. Furthermore, hemostasis seems to be disrupted due to platelet dysfunction, which is offset by increased blood flow in engorged vessels, along with a rise in the secretion of transforming growth factor β-3, leading to flawed endometrial decidualization [15]. 

The primary approach to treating AUB and HMB linked to UFs is medical therapy [16]. According to a review published by American Family Physician, the levonorgestrel-releasing intrauterine system is highly effective in reducing heavy menstrual bleeding, and is comparable to a hysterectomy in terms of quality-adjusted life years. Estrogen-progestin oral contraceptives also reduce bleeding significantly and are useful for managing bleeding in patients with ovulatory dysfunction. Continuous use of oral progestins is another hormonal option to reduce bleeding, although long-term patient satisfaction with this treatment is relatively low. Two well-tolerated, nonhormonal alternatives are oral tranexamic acid (Lysteda), and nonsteroidal anti-inflammatory drugs (NSAIDs). Both of these nonhormonal options are used only during bleeding episodes, and tranexamic acid is safe for patients trying to conceive [10]. Recent studies indicate that no single medication has been proven superior to others, although certain drugs may provide greater benefits and be more specifically tailored for managing AUB and HMB. Moreover, most of these treatments focus on the endometrium rather than the fibroids themselves, resulting in a decrease in menstrual blood flow [11]. 

In this case, the patient was initially managed with norethisterone (Ortho Micronor 0.35 mg) and subsequently Myfembree. Myfembree is a combination therapy of relugolix, estradiol, and norethindrone acetate. Relugolix is a gonadotropin-releasing hormone (GnRH) receptor antagonist that reduces serum estradiol and progesterone levels to those typically seen in postmenopausal women. The addition of estradiol and norethisterone acetate helps to mitigate bone loss and hot flashes caused by relugolix [17]. Recent literature states that relugolix combination therapy leads to a significant rapid reduction in menstrual blood loss. The therapy also helps reduce anemia and increase the rate of amenorrhea, and maintain bone mineral density [11]. 

Despite some improvement, the patient continued to experience significant pelvic pain and extended menstrual periods. The next consideration was surgical intervention. Current surgical options depend on factors such as the number, location, and size of uterine fibroids, as well as the patient’s reproductive goals or desire to preserve the uterus. Myomectomy and uterine artery embolization will preserve the uterus; however, there is still a risk for recurrence [11]. Endometrial ablation is a less invasive, lower-risk surgical option that offers effectiveness comparable to the levonorgestrel-releasing intrauterine system [18]. However, this technique primarily leads to a reduction in fibroid volume rather than effectively controlling AUB and HMB [11].

Hysterectomy has been proven to have the highest improvement in QoL [19]. The patient in this case underwent a successful supracervical hysterectomy, bilateral salpingectomy, lysis of adhesions, and left ovarian cystectomy. Postoperative histological analysis confirmed benign serous cystadenoma and leiomyomas without malignancy. The patient's recovery was uneventful, with mild residual pelvic pain. This favorable outcome reinforces the importance of a thorough surgical approach and comprehensive patient counseling.

## Conclusions

This report highlights the challenges and considerations in managing symptomatic leiomyomas, especially in patients with significant comorbidities. Pharmacological treatments, while beneficial, may not always provide complete relief, and hence surgical intervention is often required. The successful surgical outcome in this case, with benign postoperative findings, emphasizes the importance of individualized treatment planning and patient education. This approach is critical to optimizing patient outcomes and quality of life, particularly in complex cases involving multiple health conditions.
